# Bounding Seed Loss from Isolated Habitat Patches

**DOI:** 10.1007/s11538-024-01367-0

**Published:** 2024-10-28

**Authors:** Benjamin Hafner, Katherine Meyer

**Affiliations:** Mathematics and Statistics Department, Carleton College, One North College Street, Northfield, MN 55057 USA

**Keywords:** Model, Propagules, Dispersal success, Dispersal kernel, Habitat fragmentation, Spillover

## Abstract

Dispersal of propagules (seeds, spores) from a geographically isolated habitat into an uninhabitable matrix can play a decisive role in driving population dynamics. ODE and integrodifference models of these dynamics commonly feature a “dispersal success” parameter representing the average proportion of dispersing propagules that remain in viable habitat. While dispersal success can be estimated by empirical measurements or by integration of dispersal kernels, one may lack resources for fieldwork or details on dispersal kernels for numerical computation. Here we derive simple upper bounds on the proportion of propagule loss—the complement of dispersal success—that require only habitat area, habitat perimeter, and the mean dispersal distance of a propagule. Using vector calculus in a probabilistic framework, we rigorously prove bounds for the cases of both symmetric and asymmetric dispersal. We compare the bounds to simulations of integral models for the population of *Asclepias syriaca* (common milkweed) at McKnight Prairie—a 14 hectare reserve surrounded by agricultural fields in Goodhue County, Minnesota—and identify conditions under which the bounds closely estimate propagule loss.

## Introduction

This paper concerns the proportion of propagules (seeds, spores) from a population in an isolated habitat patch that disperse outside of that patch. In the right conditions, dispersal may help individuals reach environments with lower conspecific competition, fewer natural enemies, and new opportunities for establishment (Beckman and Sullivan 2023; Levin et al. 2003). Dispersal can also support metapopulation persistence despite local extinctions by allowing a species to (re)colonize patches and bet-hedge against fluctuating habitat quality (Levin et al. 2003; Hanski 2001). But for a small site surrounded by a large matrix of unviable habitat, dispersal out of the patch threatens local population persistence when emigration outweighs the arrival of propagules from external sources (Hanski 2001). Cheptou and colleagues (2008) documented a striking urban example of this dispersal risk in the weed *Crepis sancta*, whose dispersing seeds departed from tree plantings an estimated 55% of the time, resulting in selection for a nondispersing seed variant.

The proportion of propagules that leave viable habitat emerged as a key parameter for predicting population crashes in the colonization-mortality model of Cooney et al. (2022)—a reformulation of Tilman’s 1994 spatially implicit extinction debt model that includes spatial dispersal processes. In addition, the complementary probability of “dispersal success”—the probability of a propagule staying within a habitat patch—can be used to approximate steady-state solutions to integrodifference equations that model populations in continuous space with discrete growth and dispersal phases (Van Kirk and Lewis 1997; Lutscher 2019). Thus simple methods to quantify propagule loss could not only help managers assess seed movement from remnant populations but also inform more complex models to study population persistence in patchy habitats.

One empirical method to estimate propagule loss is the area-release experiment, where seeds or spores are released uniformly within a patch and then allowed to disperse. After dispersal, the fraction of propagules lying within the patch is recorded, giving the probability of dispersal success (Lutscher 2019) whose complement is propagule loss.

If such an empirical experiment is not feasible, one can also predict the probability of propagule loss analytically by integrating functions that represent the density of propagule sources and the probabilistic patterns of propagule dispersal (Cooney et al. 2022; Klausmeier 1998). This approach requires a known dispersal kernel $f_d({\varvec{v}})$, a probability density function that describes the probability of a propagule dispersing by the displacement vector ${\varvec{v}}$. The density of propagules arriving at a location ${\varvec{x}}$ in ${\mathbb {R}}^2$ is given by (Nathan et al. 2012)

1 $$\begin{aligned} \begin{array}{c} \text {propagule arrival}\\ \text {density at } {\varvec{x}} \end{array}=\iint \limits _{\text {habitat}} \left( \begin{array}{c} \text {propagule} \\ \text {source density}\\ \text {at }{\varvec{x}}_p \end{array} \right) \left( \begin{array}{c} \text {dispersal kernel}\\ \text {evaluated at} \\ {\varvec{x}}-{\varvec{x}}_p \end{array} \right) \, dA \end{aligned}$$

and total propagule loss can be obtained by integrating propagule arrival density over the complement of the habitat patch:

2 $$\begin{aligned} \text {propagule loss }=\iint \limits _{\begin{array}{c} \text {not} \\ \text {habitat} \end{array}} \left( \begin{array}{c} \text {propagule}\\ \text {arrival density} \end{array}\right) \ dA. \end{aligned}$$

While this approach does not explicitly require field work like an area-release experiment, it does assume detailed knowledge of the habitat geometry and dispersal kernel. In many studies, knowledge of these attributes may be incomplete.

**Fig. 1:**
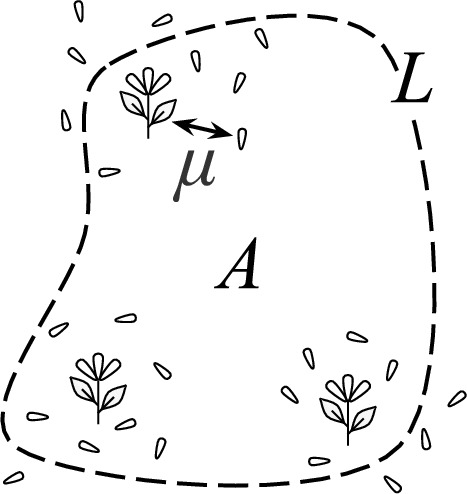
Schematic of quantities used to bound seed loss, with the habitat boundary depicted as a dashed line

Here we derive upper bounds for propagule loss that could be especially useful when one has only rough knowledge of the habitat geometry and dispersal kernel.

For brevity, we refer to propagule sources as plants and propagules as seeds. We make the simplifying assumptions that

(i) plants are uniformly distributed within a viable habitat and
(ii) the dispersal kernel associated with a plant is independent from its location in the habitat.

In this context, we consider the probability *p* that a single arbitrary seed is lost, which well approximates the proportion of seeds lost from a large population. Under the above assumptions, this probability of seed loss *p* from a population in an isolated habitat patch is bounded by

3 $$\begin{aligned} p\le \frac{\mu L}{2A} \end{aligned}$$

where *A* is the habitat area, *L* is the perimeter, and $\mu$ is the mean seed dispersal distance (illustrated in Fig. 1). If in addition we assume that dispersal patterns are identical in each direction—that is, rotationally symmetric—then we can decrease this upper bound to

4 $$\begin{aligned} p\le \frac{\mu L}{\pi A}. \end{aligned}$$

Given that patch area *A* appears in the denominator of these expressions, while patch perimeter *L* appears in the numerator, we expect the seed loss bound to decrease as we scale up a habitat region while maintaining its shape (since *L* grows linearly while *A* grows quadratically). Loosely speaking, the ratio *A*/*L* can be interpreted as the habitat’s characteristic length scale, which must be kept larger than $\mu$ in order to limit seed loss.

In addition, we observe that these bounds are tight, in two senses. In a practical sense, when applied to an ecologically relevant plant population, the bounds are close to the true value of *p*, especially in the rotationally symmetric case when the habitat is large relative to the mean dispersal distance (see Sect. 5). And from a mathematical perspective, they are tight in the sense that the constant factors 1/2 and $1/\pi$ cannot be improved (see Appendix B).

It is interesting to note that the bound $\frac{\mu L}{\pi A}$ on the proportion of seed loss out of a habitat patch in the case of symmetric dispersal also appears in Chisholm and Lichstein’s (2009) approximation to the immigration parameter *m* in the neutral theory of biodiversity. In Appendix A, we demonstrate that *m* and *p* are in fact equivalent. In light of this equivalence, our work makes two major new contributions. First, we provide a rigorous proof that $\frac{\mu L}{\pi A}$ is a strict upper bound, not just an approximation, for the proportion of seed loss (or immigration, in the case of *m*). Our proof is grounded in the language of random variables and vector calculus and avoids heuristic arguments. Second, using the these tools we are able to handle the novel case of asymmetric dispersal, yielding the bound $\frac{\mu L}{2 A}$ on both *p* and *m*.

The paper is organized as follows. We begin by establishing a probabilistic modeling framework to precisely define *p* in Sect. 2. Then, in Sect. 3, we translate the spatial geometry of seed movement from dispersal kernels to vector fields and use the divergence theorem to reframe seed loss as a flux of seeds across the habitat boundary. In Sect. 4 we bound seed flux across the habitat boundary. We put all the pieces together in Theorem 7, which formalizes the claims (3) and (4). Once the proof of Theorem 7 is complete, we provide an example of how it could be applied in a conservation context in Sect. 5. Specifically, we compute seed loss bounds for a population of *Ascelpias syriaca* (common milkweed) located at McKnight Prairie, an isolated reserve surrounded by agriculture, and show it closely matches an integral model of seed loss. In Sect. 6 we discuss methods for estimating mean dispersal distance as well as future theoretical directions.

## Modeling Framework

Throughout this text, given sets *U* and $V\subset {\mathbb {R}}^2$ we use $\partial U$ to denote the boundary of *U*, $U^c$ for set complement, and $U\backslash V$ for the set difference $U\cap V^c$.

We represent viable habitat for a plant population of interest as an open subset $H\subset {\mathbb {R}}^2$ with finite area *A* and perimeter *L*. We treat plant positions, seed displacements, and seed landing locations as continuous random vectors with values in ${\mathbb {R}}^2$.

The random vector ${\textbf{X}}_p$ models the position of plants in the population. Recall that ${\textbf{X}}_p$ relates to its probability density function $f_p$ as follows: given a set $U\subset {\mathbb {R}}^2$,

5 $$\begin{aligned} P({\textbf{X}}_p\in U)=\iint \limits _{{\varvec{x}}\in U} f_p({\varvec{x}}) \, dA. \end{aligned}$$

Here *dA* refers to an infinitesimal area of integration (e.g. $dx_1 dx_2$) and does not imply a relationship to total habitat area *A*. We assume plant positions are uniformly distributed over *H*, so

6 $$\begin{aligned} f_p({\varvec{x}})={\left\{ \begin{array}{ll}1/ A & \text {if } {\varvec{x}}\in H \\ 0 & \text {otherwise} \end{array}\right. } \end{aligned}$$

The seeds released by a plant are displaced by a random dispersal vector ${\textbf{X}}_d$ from the source plant before they land. The probability density function $f_d$ associated with ${\textbf{X}}_d$ is also known as a dispersal kernel (Nathan et al. 2012). Importantly, we assume ${\textbf{X}}_p$ and ${\textbf{X}}_d$ are independent: the position of a source plant within the habitat does not influence the dispersal pattern of its seeds (see Sect. 6 for discussion of this assumption). The mean dispersal distance of seeds is given by

7 $$\begin{aligned} \mu =E[ \, |{\textbf{X}}_d| \, ]=\iint \limits _{{\varvec{x}}\in {\mathbb {R}}^2} |{\varvec{x}}|f_d({\varvec{x}}) \, dA. \end{aligned}$$

We assume that $f_d$ decays rapidly enough as $|{\varvec{x}}|\rightarrow \infty$ to make $\mu$ well-defined. Commonly used functional forms of $f_d$ include the inverse Gaussian for wind-dispersed species and the exponential power function (Bullock et al. 2017; Nathan et al. 2012).

**Fig. 2:**
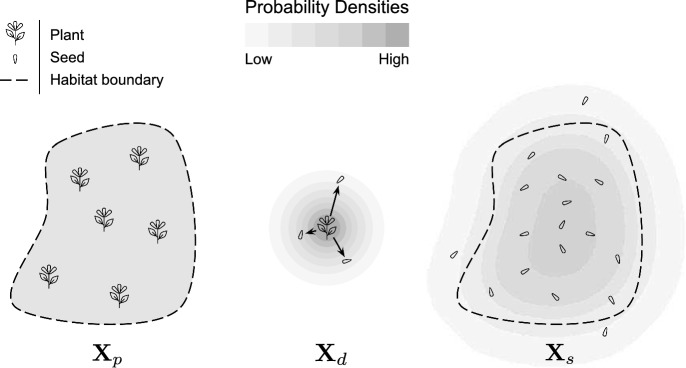
Visualization of the probability densities associated with random variables for plant locations ${\textbf{X}}_p$, seed dispersal ${\textbf{X}}_d$, and seed landing locations ${\textbf{X}}_s$

We define the random vector ${\textbf{X}}_s$ by

8 $$\begin{aligned} {\textbf{X}}_s= {\textbf{X}}_p+ {\textbf{X}}_d\end{aligned}$$

to model the landing position of a seed that originated from the plant population in *H*. We assume that $H^c$ is not viable habitat for the population of interest, so seeds that originate in *H* but land in $H^c$ are lost. Our main quantity of interest, the probability *p* of seed loss from the habitat, is given by

9 $$\begin{aligned} p=P({\textbf{X}}_s\in H^c)=\iint \limits _{{\varvec{x}}\in H^c}f_s({\varvec{x}}) \, dA \end{aligned}$$

where $f_s$ is the probability density of ${\textbf{X}}_s$. Figure 2 gives a visual summary of the random variables ${\textbf{X}}_p$, ${\textbf{X}}_d$, and ${\textbf{X}}_s$.

As a first step towards bounding seed loss probability *p*, we unpack *p*’s defining integral from (9) in terms of habitat area *A* and dispersal kernel $f_d$.

### Proposition 1

The seed loss probability *p* defined in Eq. (9) is equivalent to

10 $$\begin{aligned} p=\frac{1}{A}\iint \limits _{{\varvec{x}}_p\in H} \ \ \iint \limits _{{\varvec{x}}\in H^c} f_d({\varvec{x}}- {\varvec{x}}_p) \, dA \, dA. \end{aligned}$$

### Proof

Because ${\textbf{X}}_p$ and ${\textbf{X}}_d$ are independent, the density of their sum ${\textbf{X}}_s$ is the convolution of their densities:

11 $$\begin{aligned} f_s({\varvec{x}})=\iint \limits _{{\varvec{x}}_p\in {\mathbb {R}}^2}f_p({\varvec{x}}_p)f_d({\varvec{x}}-{\varvec{x}}_p) \, dA. \end{aligned}$$

Replacing $f_p$ in (11) with its definition from (6) yields

12 $$\begin{aligned} f_s({\varvec{x}})=\frac{1}{A}\iint \limits _{{\varvec{x}}_p\in H}f_d({\varvec{x}}-{\varvec{x}}_p) \, dA. \end{aligned}$$

By substituting $f_s$ from (12) into the definition (9) of *p* and reversing the order of integration, one obtains the expression (10). $\square$

The representation of *p* in Proposition 1 can be interpreted as seed loss probability for an individual plant at ${\varvec{x}}_p$ (the inner integral), averaged uniformly over the habitat *H* (the outer integral). This form is useful for calculating *p* numerically from a known habitat *H* and dispersal kernel $f_d$, as done by Cooney et al. (2022).

## Seed Loss as the Flux of a Vector Field Across Habitat Boundary

To bound *p* in terms of habitat perimeter *L*, we transform the inner integral over $H^c$ in Eq. (10) into a line integral around the habitat boundary $\partial H$. Specifically, we express seed loss as a flux of seeds crossing the habitat boundary. To do so, we must describe seed flow in the language of vector fields. Section 3.1 constructs a vector field describing dispersal from a single plant and Sect. 3.2 integrates over all plant locations to obtain a vector field representing total seed dispersal.

### Vector Field of a Single Plant

**Fig. 3:**
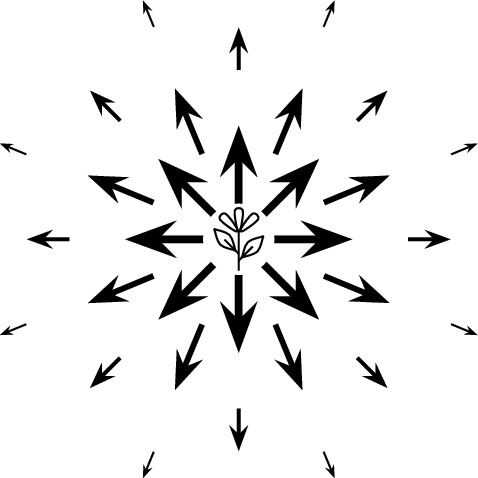
The vector field ${\textbf{F}}$ represents the spatial flow of seeds away from a source plant

The dispersal of seeds from a single plant is typically represented as a probability density function $f_d$, but the same information can also be represented as a vector field, which we call ${\textbf{F}}$ (Fig. 3). The vector field ${\textbf{F}}$ describes the motion of seeds as they disperse—like a flowing fluid—whereas $f_d$ describes their final resting positions.

The mathematical link between ${\textbf{F}}$ and $f_d$ is the divergence operator. Intuitively, a positive divergence of ${\textbf{F}}$ indicates a source (seeds being released) and a negative divergence indicates a sink (seeds landing). Our definition of ${\textbf{F}}$ below yields a positive divergence singularity at the origin, representing the plant as a point source of seeds, and yields $\nabla \cdot {\textbf{F}}= -f_d$ everywhere else, encoding seed landing positions. For more physical interpretation and motivation of ${\textbf{F}}$’s definition, see Appendix C.

#### Definition 1

Given a dispersal kernel $f_d$, the corresponding vector field ${\textbf{F}}$ is

13 $$\begin{aligned} {\textbf{F}}({\varvec{x}})=\frac{{\varvec{x}}}{r_x^2}\int \limits _{r_x}^\infty f_d(r,\theta _x) \, r \, dr. \end{aligned}$$

#### Lemma 2

For all non-zero ${\varvec{x}}\in {\mathbb {R}}^2$,

14 $$\begin{aligned} \nabla \cdot {\textbf{F}}({\varvec{x}})=-f_d({\varvec{x}}). \end{aligned}$$

#### Proof

We work in polar coordinates with unit vectors ${\varvec{{\hat{r}}}}={\varvec{x}}/|{\varvec{x}}|$ and ${\varvec{{\hat{\theta }}}}$ the rotation of ${\varvec{{\hat{r}}}}$ by $\pi /2$. In these coordinates, ${\textbf{F}}({\varvec{x}})=|{\textbf{F}}({\varvec{x}})|{\varvec{{\hat{r}}}}+0\,{\varvec{{\hat{\theta }}}}$ and by the polar divergence formula

15 $$\begin{aligned} \nabla \cdot {\textbf{F}}&=\frac{1}{r_x}\frac{\partial }{\partial r_x}\Big [r_x |{\textbf{F}}| \Big ]+\frac{1}{r_x}\frac{\partial }{\partial \theta }\Big [0\Big ] \end{aligned}$$

16 $$\begin{aligned}&=\frac{1}{r_x}\frac{\partial }{\partial r_x}\Big [\int \limits _{r_x}^\infty f_d(r,\theta _x) \, r \, dr\Big ] \end{aligned}$$

17 $$\begin{aligned}&=\frac{1}{r_x}\big (-f_d(r_x,\theta _x)r_x\big ) \end{aligned}$$

18 $$\begin{aligned}&=-f_d(r_x,\theta _x). \end{aligned}$$

$$\square $$

Using Definition 1 and Lemma 2, one can convert from a dispersal kernel $f_d$ to a vector field ${\textbf{F}}$ and back again. The two objects carry the same information about seed dispersal, simply in different forms.

The remainder of Sect. 3.1 concerns the flux of ${\textbf{F}}$ across the habitat boundary, which represents a single plant’s probability of seed loss.

#### Lemma 3

Let *D* be a disk shaped habitat with a single plant at its center, the origin. Then the flux of ${\textbf{F}}$ across the habitat boundary $\partial D$ is equal to the probability that the plant’s seeds are lost (land outside *D*). That is,

19 $$\begin{aligned} \oint \limits _{{\varvec{x}}\in \partial D} {\textbf{F}}({\varvec{x}})\cdot {\textbf{n}}\, ds = \iint \limits _{{\varvec{x}}\in D^c} f_d({\varvec{x}}) \, dA \end{aligned}$$

where ${\textbf{n}}$ is the outward pointing unit vector normal to the boundary.

#### Proof

Suppose the disk has radius *R*. Then applying Definition 1 and simplifying yields

20 $$\begin{aligned} \oint \limits _{{\varvec{x}}\in \partial D}{\textbf{F}}({\varvec{x}})\cdot {\textbf{n}}\, ds&=\oint \limits _{{\varvec{x}}\in \partial D} \frac{1}{R}\int \limits _{R}^\infty f_d(r,\theta _x) \, r \, dr \, ds. \end{aligned}$$

Replacing *ds* with $R \, d\theta _x$ and parameterizing the loop integral by $\theta _x$ gives

21 $$\begin{aligned} \oint \limits _{{\varvec{x}}\in \partial D}{\textbf{F}}({\varvec{x}})\cdot {\textbf{n}}\, ds&=\int \limits _{0}^{2\pi } \frac{1}{R}\int \limits _{R}^\infty f_d(r,\theta _x) \, r \, dr \, R \, d\theta _x \end{aligned}$$

22 $$\begin{aligned}&=\int \limits _{0}^{2\pi } \int \limits _{R}^\infty f_d(r,\theta _x) \, r \, dr \, d\theta _x \end{aligned}$$

23 $$\begin{aligned}&=\iint \limits _{{\varvec{x}}\in D^c} f_d({\varvec{x}}) \, dA. \end{aligned}$$

$$\square $$

In Proposition 4, we use the divergence theorem to generalize Lemma 3 from a disk *D* to a generic habitat shape *H* and allow the plant to reside at any point ${\varvec{x}}_p$ in *H*, not just the origin.

#### Proposition 4

For all $${\varvec{x}}_p\in H$$,

24 $$\begin{aligned} \iint \limits _{{\varvec{x}}\in H^c} f_d({\varvec{x}}- {\varvec{x}}_p) \, dA=\oint \limits _{{\varvec{x}}\in \partial H} {\textbf{F}}({\varvec{x}}- {\varvec{x}}_p) \cdot {\textbf{n}}\, ds. \end{aligned}$$

#### Proof

**Fig. 4:**
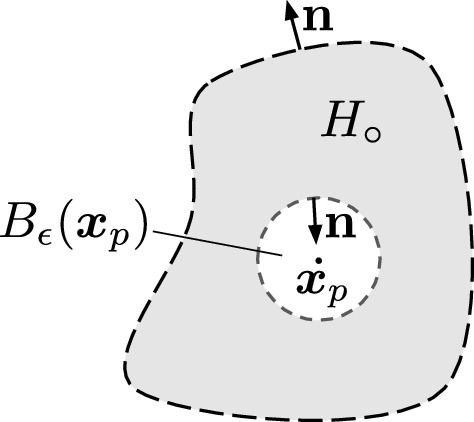
Orientation of normal vectors used in the proof of Proposition 4

We begin with the right hand side and derive the left. To apply the divergence theorem, care is required around the singularity of ${\textbf{F}}({\varvec{x}}-{\varvec{x}}_p)$ when ${\varvec{x}}={\varvec{x}}_p$. Fix ${\varvec{x}}_p\in H$ and choose $\epsilon >0$ such that the ball $B_\epsilon ({\varvec{x}}_p)$ of radius $\epsilon$ around ${\varvec{x}}_p$ lies entirely within *H*. Let $H_\circ =H\backslash B_\epsilon ({\varvec{x}}_p)$. Note that the boundary of $H_\circ$ consists of the boundary of *H* and the boundary of $B_\epsilon ({\varvec{x}}_p)$. Let ${\textbf{n}}$ represent the unit normal to each boundary, with orientations shown in Fig. 4. We have that

25 $$\begin{aligned} \oint \limits _{{\varvec{x}}\in \partial H}{\textbf{F}}({\varvec{x}}-{\varvec{x}}_p)\cdot {\textbf{n}}\, ds =\underbrace{\oint \limits _{{\varvec{x}}\in \partial H_\circ }{\textbf{F}}({\varvec{x}}-{\varvec{x}}_p)\cdot {\textbf{n}}\ ds }_{\text {I}} -\underbrace{\oint \limits _{\underset{\partial B_\epsilon ({\varvec{x}}_p)}{{\varvec{x}}\in }} {\textbf{F}}({\varvec{x}}-{\varvec{x}}_p)\cdot {\textbf{n}}\, ds}_{\text {II}}.\nonumber \\ \end{aligned}$$

Because $H_\circ$ excludes the singularity at ${\varvec{x}}_p$, we may apply the divergence theorem to integral I of (25). This gives

26 $$\begin{aligned} \begin{aligned} \oint \limits _{{\varvec{x}}\in \partial H_\circ }{\textbf{F}}({\varvec{x}}-{\varvec{x}}_p)\cdot {\textbf{n}}\ ds&= \iint \limits _{{\varvec{x}}\in H_\circ } \nabla \cdot {\textbf{F}}({\varvec{x}}-{\varvec{x}}_p) \, dA\\ &= -\iint \limits _{{\varvec{x}}\in H_\circ } f_d({\varvec{x}}-{\varvec{x}}_p) \, dA, \end{aligned} \end{aligned}$$

where the second equality follows from Lemma 2. Integral II is handled by Lemma 3, since $B_\epsilon ({\varvec{x}}_p)$ is a disk:

27 $$\begin{aligned} \oint \limits _{{\varvec{x}}\in \partial B_\epsilon ({\varvec{x}}_p)} {\textbf{F}}({\varvec{x}}-{\varvec{x}}_p)\cdot {\textbf{n}}\, ds = -\iint \limits _{{\varvec{x}}\in B_\epsilon ({\varvec{x}}_p)^c} f_d({\varvec{x}}-{\varvec{x}}_p) \, dA. \end{aligned}$$

Note the translation from ${\varvec{x}}$ in Lemma 3 to ${\varvec{x}}- {\varvec{x}}_p$ in Eq. (27), along with the re-orientation of the unit normal from outward to inward, resulting in negation.

Substituting (26) and (27) for integrals I and II in (25) gives

28 $$\begin{aligned} \oint \limits _{{\varvec{x}}\in \partial H}{\textbf{F}}({\varvec{x}}-{\varvec{x}}_p)\cdot {\textbf{n}}\, ds = -\iint \limits _{{\varvec{x}}\in H_\circ } f_d({\varvec{x}}-{\varvec{x}}_p) \, dA+\iint \limits _{{\varvec{x}}\in B_\epsilon ({\varvec{x}}_p)^c} f_d({\varvec{x}}-{\varvec{x}}_p) \, dA. \end{aligned}$$

Because $H_\circ \subset B_\epsilon ({\varvec{x}}_p)^c$, the difference of integrals in (28) is the integral over their set difference $B_\epsilon ({\varvec{x}}_p)^c\backslash H_\circ$, which is $H^c$. This yields our desired result

$$\begin{aligned} \oint \limits _{{\varvec{x}}\in \partial H} {\textbf{F}}({\varvec{x}}- {\varvec{x}}_p) \cdot {\textbf{n}}\, ds=\iint \limits _{{\varvec{x}}\in H^c} f_d({\varvec{x}}- {\varvec{x}}_p) \, dA. \end{aligned}$$

$$\square $$

### Integrating Seed Flux from All Plant Locations

**Fig. 5:**
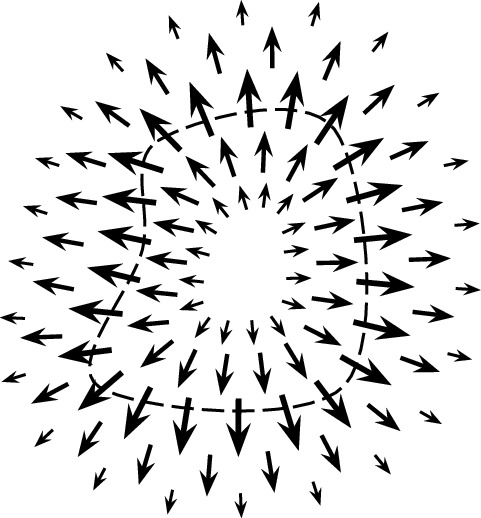
The total dispersal field ${\textbf{G}}$ models the net flow of all seeds originating from the plant population in *H* (Eq. (31)). In the middle of a large habitat ${\textbf{G}}$ tends to be small, because seed movement in all directions results in a net movement near zero, unless prevailing winds or some other factor biases seed dispersal in a specific direction. As one moves far outside the habitat where few seeds reach, ${\textbf{G}}$ also tends to zero. It is near the edge of the habitat, where plant density changes abruptly, that ${\textbf{G}}$ is typically largest and points outward. The outward flux of ${\textbf{G}}$ across the habitat boundary, depicted here as a dotted line, represents seed loss (Eq. (32))

Next we develop a second vector field ${\textbf{G}}$ that aggregates seed movement from all plants in the habitat (Fig. 5). Using Proposition 4, we can rewrite the inner integral in the expression (10) for seed loss probability *p* in terms of seed flux from a single source at ${\varvec{x}}_p$ across the habitat boundary. This gives

29 $$\begin{aligned} p=\frac{1}{A}\iint \limits _{{\varvec{x}}_p\in H} \ \oint \limits _{{\varvec{x}}\in \partial H} {\textbf{F}}({\varvec{x}}-{\varvec{x}}_p)\cdot {\textbf{n}}\, ds \, dA. \end{aligned}$$

Reversing the order of integration and moving ${\textbf{n}}$ out of the inner integral yields

30 $$\begin{aligned} p&=\frac{1}{A}\oint \limits _{{\varvec{x}}\in \partial H} \ \iint \limits _{{\varvec{x}}_p\in H} {\textbf{F}}({\varvec{x}}-{\varvec{x}}_p) \, dA \, \cdot {\textbf{n}}\, ds. \end{aligned}$$

The inner integral in (30) now represents the effective flow of seeds contributed from all plants in the habitat at a point ${\varvec{x}}$ on the habitat boundary. We call this the total dispersal field and denote it

31 $$\begin{aligned} {\textbf{G}}({\varvec{x}})\equiv \displaystyle \iint \limits _{{\varvec{x}}_p\in H} {\textbf{F}}({\varvec{x}}- {\varvec{x}}_p) \, dA, \end{aligned}$$

so that

32 $$\begin{aligned} p=\frac{1}{A}\oint \limits _{{\varvec{x}}\in \partial H} {\textbf{G}}({\varvec{x}})\cdot {\textbf{n}}\, ds. \end{aligned}$$

## Bounding Seed Loss

Based on Eq. (32), in order to bound *p* it suffices to bound the magnitude of ${\textbf{G}}\cdot {\textbf{n}}$. We derive two upper bounds on ${\textbf{G}}\cdot {\textbf{n}}$: one with no assumptions on the symmetry of the dispersal kernel $f_d$ (Lemma 5) and another when $f_d$ is rotationally symmetric (Lemma 6).

### Lemma 5

For all ${\varvec{x}}\in {\mathbb {R}}^2$ and for all unit vectors ${\textbf{n}}\in {\mathbb {R}}^2$,

$$\begin{aligned} {\textbf{G}}({\varvec{x}})\cdot {\textbf{n}}\le \frac{{\textbf{n}}}{2}\cdot E({\textbf{X}}_d)+\frac{\mu }{2}. \end{aligned}$$

### Proof

It follows from the definition (31) of ${\textbf{G}}$ that

$$\begin{aligned} {\textbf{G}}({\varvec{x}})\cdot {\textbf{n}}= \iint \limits _{{\varvec{x}}_p\in H} {\textbf{F}}({\varvec{x}}-{\varvec{x}}_p)\cdot {\textbf{n}}\, dA. \end{aligned}$$

To simplify the following argument, we define ${\varvec{y}}={\varvec{x}}-{\varvec{x}}_p$ as a shorthand for the seed dispersal vector. Additionally, let $\hat{H}=\{{\varvec{y}}\in {\mathbb {R}}^2 \, | \, {\varvec{x}}-{\varvec{y}}\in H\}$ so that

$$\begin{aligned} {\textbf{G}}({\varvec{x}})\cdot {\textbf{n}}= \iint \limits _{{\varvec{y}}\in \hat{H}} {\textbf{F}}({\varvec{y}})\cdot {\textbf{n}}\, dA. \end{aligned}$$

Expanding ${\textbf{F}}$ by Definition 1, we have

33 $$\begin{aligned} {\textbf{G}}({\varvec{x}})\cdot {\textbf{n}}= \iint \limits _{{\varvec{y}}\in \hat{H}} \frac{{\varvec{y}}\cdot {\textbf{n}}}{r_y^2}\int \limits _{r_y}^\infty f_d(r,\theta _y) \, r \, dr \, dA \end{aligned}$$

where $\theta _y, r_y$ are the polar coordinates of ${\varvec{y}}$. Next, we use the fact that ${\varvec{y}}\cdot {\textbf{n}}\le \frac{{\varvec{y}}\cdot {\textbf{n}}}{2} + \frac{|{\varvec{y}}\cdot {\textbf{n}}|}{2}$ to produce the inequality

34 $$\begin{aligned} {\textbf{G}}({\varvec{x}})\cdot {\textbf{n}}\le \iint \limits _{{\varvec{y}}\in \hat{H}} \left( \frac{{\varvec{y}}\cdot {\textbf{n}}}{2} + \frac{|{\varvec{y}}\cdot {\textbf{n}}|}{2}\right) \frac{1}{r_y^2}\int \limits _{r_y}^\infty f_d(r,\theta _y) \, r \, dr \, dA. \end{aligned}$$

Substituting $\frac{{\varvec{y}}\cdot {\textbf{n}}}{2} + \frac{|{\varvec{y}}\cdot {\textbf{n}}|}{2}$ for ${\varvec{y}}\cdot {\textbf{n}}$ has no effect when ${\varvec{y}}\cdot {\textbf{n}}>0$, that is, when the seed dispersal vector points outward across the habitat boundary. But when ${\varvec{y}}\cdot {\textbf{n}}<0$, it is replaced by zero. Thus, we ignore the contribution of any seeds flowing inward across the habitat boundary. Although at first it appears to introduce needless complication, this substitution enables the rest of the proof.

Because $\frac{{\varvec{y}}\cdot {\textbf{n}}}{2} + \frac{|{\varvec{y}}\cdot {\textbf{n}}|}{2}$ is never negative, we are now free to expand the region of integration from ${\varvec{y}}\in \hat{H}$ to all ${\varvec{y}}\in {\mathbb {R}}^2$, which we parameterize by $\theta _y, r_y$, with $\theta _y$ measured relative to ${\textbf{n}}$ so that ${\varvec{y}}\cdot {\textbf{n}}=r_y\cos \theta _y$. With this, (34) becomes

35 $$\begin{aligned} {\textbf{G}}({\varvec{x}})\cdot {\textbf{n}}&\le \int \limits _0^{2\pi }\int \limits _0^\infty \left( \frac{r_y\cos \theta _y}{2}+\frac{|r_y\cos \theta _y|}{2}\right) \frac{1}{r_y^2}\int \limits _{r_y}^\infty f_d(r,\theta _y) \, r \, dr \, r_y \, dr_y \, d\theta _y \end{aligned}$$

36 $$\begin{aligned}&=\int \limits _0^{2\pi }\int \limits _0^\infty \int \limits _{r_y}^\infty \left( \frac{\cos \theta _y}{2}+\frac{|\cos \theta _y|}{2}\right) f_d(r,\theta _y) \, r \, dr \, dr_y \, d\theta _y. \end{aligned}$$

We interchange the order of integration between *dr* and $dr_y$ to obtain

37 $$\begin{aligned} {\textbf{G}}({\varvec{x}})\cdot {\textbf{n}}\le \int \limits _0^{2\pi } \int \limits _0^\infty \int \limits _0^r \left( \frac{\cos \theta _y}{2}+\frac{|\cos \theta _y|}{2}\right) f_d(r,\theta _y) \, r \, dr_y \, dr \, d\theta _y \end{aligned}$$

and evaluate the innermost integral, yielding an additional factor of *r*:

38 $$\begin{aligned} {\textbf{G}}({\varvec{x}})\cdot {\textbf{n}}\le \int \limits _0^{2\pi }\ \int \limits _0^\infty \left( \frac{r\cos \theta _y}{2}+\frac{r|\cos \theta _y|}{2}\right) f_d(r,\theta _y) \, r \, dr \, d\theta _y. \end{aligned}$$

Let ${\hat{{\varvec{y}}}}=\frac{r}{r_y}{\varvec{y}}$, so that ${\hat{{\varvec{y}}}}$ has the same direction as ${\varvec{y}}$ but magnitude *r* instead of $r_y$. Then $r\cos \theta _y=\hat{\varvec{y}} \cdot {{\textbf {n}}}$ and

39 $$\begin{aligned} {{\textbf {G}}}({\varvec{x}})\cdot {{\textbf {n}}}&\le \int \limits _0^{2\pi }\int \limits _0^\infty \left( \frac{{\hat{\varvec{y}}\cdot {{\textbf {n}}}}}{2}+\frac{|{\hat{\varvec{y}}\cdot {{\textbf {n}}}}|}{2}\right) f_d(r,\theta _y) \, r \, dr \, d\theta _y \end{aligned}$$

40 $$\begin{aligned}&=\iint \limits _{{\hat{{\varvec{y}}}}\in {\mathbb {{R}}^2}} \left( \frac{{\hat{\varvec{y}}\cdot {{\textbf {n}}}}}{2}+\frac{|{\hat{\varvec{y}}\cdot {{\textbf {n}}}}|}{2}\right) f_d({\hat{{\varvec{y}}}}) \, dA \end{aligned}$$

41 $$\begin{aligned}&\le \frac{{\textbf{n}}}{2}\cdot \iint \limits _{{\hat{{\varvec{y}}}}\in {\mathbb {{R}}^2}}{\hat{{\varvec{y}}}} \, f_d({\hat{{\varvec{y}}}}) \, dA + \frac{1}{2}\iint \limits _{{\hat{{\varvec{y}}}}\in {\mathbb {{R}}^2}} \big |{\hat{{\varvec{y}}}}\big |f_d({\hat{{\varvec{y}}}}) \, dA \end{aligned}$$

42 $$\begin{aligned}&=\frac{{\textbf{n}}}{2}\cdot E({\textbf{X}}_d)+\frac{1}{2}\mu \end{aligned}$$

as claimed. $$\square $$

The bound in Lemma 5 remains valid when seed dispersal is asymmetric or biased in one direction, for example by prevailing winds. In contrast, Lemma 6 requires that dispersal patterns are identical in each direction, which yields a stronger bound.

### Lemma 6

Suppose $f_d$ is rotationally symmetric; that is, there exists a function ${\hat{f_d}}$ such that $f_d(r,\theta ) = {\hat{f_d}}(r)$ for all $\theta$. Then for all ${\varvec{x}}\in {\mathbb {R}}^2$ and for all unit vectors ${\textbf{n}}\in {\mathbb {R}}^2$,

$$\begin{aligned} {\textbf{G}}({\varvec{x}})\cdot {\textbf{n}}\le \frac{\mu }{\pi }. \end{aligned}$$

### Proof

Following the proof of Lemma 5 to Eq. (38), we have

$$\begin{aligned} {\textbf{G}}({\varvec{x}})\cdot {\textbf{n}}\le \int \limits _0^{2\pi }\ \int \limits _0^\infty \left( \frac{r\cos \theta _y}{2}+\frac{r|\cos \theta _y|}{2}\right) f_d(r,\theta _y) \, r \, dr \, d\theta _y. \end{aligned}$$

But because $f_d(r,\theta ) = {\hat{f_d}}(r)$, which depends only on *r*, this double integral can now be separated:

43 $$\begin{aligned} {\textbf{G}}({\varvec{x}})\cdot {\textbf{n}}&\le \int \limits _0^{2\pi } \left( \frac{\cos \theta _y}{2}+\frac{|\cos \theta _y|}{2}\right) d\theta _y \int \limits _0^\infty {\hat{f_d}}(r) \, r^2 \, dr \end{aligned}$$

44 $$\begin{aligned}&= 2 \int \limits _0^\infty {\hat{f_d}}(r) \, r^2 \, dr. \end{aligned}$$

When the definition of $\mu$ from Eq. (7) is re-written in polar coordinates,

45 $$\begin{aligned} \mu = \int \limits _0^{2\pi } \int \limits _0^\infty r {\hat{f_d}}(r) \, r \, dr \, d\theta = 2\pi \int \limits _0^\infty {\hat{f_d}}(r) \, r^2 \, dr, \end{aligned}$$

it becomes clear, upon comparison of Eqs. (44) and (45), that

46 $$\begin{aligned} {\textbf{G}}({\varvec{x}})\cdot {\textbf{n}}\le \frac{\mu }{\pi }. \end{aligned}$$

$$\square $$

### Theorem 7

Suppose a plant population with mean seed dispersal distance $\mu$ is uniformly distributed over a habitat patch *H* with finite area *A* and perimeter *L*. If dispersal is independent of plant position, then the probability that a given seed disperses outside the habitat is bounded by

$$\begin{aligned} p \le \frac{\mu L}{2 A}. \end{aligned}$$

If in addition dispersal is rotationally symmetric, then the stronger bound

$$\begin{aligned} p \le \frac{\mu L}{\pi A} \end{aligned}$$

holds.

### Proof

From Eq. (32) we have

$$\begin{aligned} p = \frac{1}{A} \oint \limits _{{\varvec{x}}\in \partial H} {\textbf{G}}({\varvec{x}})\cdot {\textbf{n}}({\varvec{x}}) \, ds, \end{aligned}$$

where ${\textbf{n}}(x)$ is the outward-oriented unit vector orthogonal to $\partial H$ at ${\varvec{x}}$. In the general case, Lemma 5 gives that

$$\begin{aligned} {\textbf{G}}({\varvec{x}})\cdot {\textbf{n}}({\varvec{x}}) \le \frac{{\textbf{n}}({\varvec{x}})}{2}\cdot E({\textbf{X}}_d)+\frac{\mu }{2}, \end{aligned}$$

and so

47 $$\begin{aligned} p&\le \frac{1}{A} \oint \limits _{{\varvec{x}}\in \partial H} \left( \frac{{\textbf{n}}({\varvec{x}})}{2}\cdot E({\textbf{X}}_d)+\frac{\mu }{2}\right) ds \end{aligned}$$

48 $$\begin{aligned}&=\frac{1}{A}\left( \frac{1}{2}E({\textbf{X}}_d) \, \cdot \oint \limits _{{\varvec{x}}\in \partial H} {\textbf{n}}({\varvec{x}}) \, ds + \oint \limits _{{\varvec{x}}\in \partial H}\frac{\mu }{2} \, ds \right) . \end{aligned}$$

The integral of ${\textbf{n}}({\varvec{x}})$ around the closed curve $\partial H$ vanishes, leaving

49 $$\begin{aligned} p \le \frac{1}{A} \oint \limits _{{\varvec{x}}\in \partial H}\frac{\mu }{2} \, ds = \frac{\mu L}{2A} \end{aligned}$$

as claimed.

In the special case that dispersal is rotationally symmetric, we have from Lemma 6 that

$$\begin{aligned} {\textbf{G}}({\varvec{x}})\cdot {\textbf{n}}\le \frac{\mu }{\pi } \end{aligned}$$

which immediately gives

$$\begin{aligned} p\le \frac{1}{A} \oint \limits _{{\varvec{x}}\in \partial H} \frac{\mu }{\pi } \, ds =\frac{\mu L}{\pi A}. \end{aligned}$$

$$\square $$

In Appendix B we show that the constant factors 1/2 and $1/\pi$ appearing in Theorem 7 are the best that can be achieved under the assumptions of this paper.

## Example

To illustrate a practical application of Theorem 7, we turn to McKnight Prairie, a 14 hectare (34 acre) patch of remnant prairie in southern Minnesota. The site was mostly spared from agricultural use due to its hilly terrain, and is now protected under the Minnesota Department of Natural Resources (DNR) Native Prairie Bank Program.

Since the plot is embedded in a matrix of commercial agriculture, any seeds that land outside its borders are effectively lost. Especially for a species like *Ascelpias syriaca* (common milkweed), whose seeds are easily carried away on the wind, one might wonder if a large fraction of seeds are lost to the surrounding farmland, hampering the species’ ability to reach available sites within the local prairie and potentially leading to population decline. In this type of scenario, Theorem 7 may provide a rough estimate and upper bounds on seed loss. We compute these bounds in Sect. 5.1, simulate seed loss via a full dispersal kernel model in Sect. 5.2, and compare the bounds to simulations in Sect. 5.3.

### Upper Bounds on Seed Loss

To compute the bounds given by Theorem 7, three inputs are required: habitat area *A*, habitat perimeter *L*, and mean dispersal distance $\mu$. We traced the border of McKnight Prairie from Google Maps and then used a simple Python program to estimate its area and perimeter: $A = 137,000$ $\hbox {m}^2$ (13.7 hectares) and $L = 1,930$ m. One could also make physical measurements or use GIS software.

Mean dispersal distance is somewhat more challenging to estimate. Section 6.1 discusses several practical routes to computing $\mu$, including the “ballistic” model for wind-dispersed seeds,

50 $$\begin{aligned} \mu =\frac{v_wh}{v_T}, \end{aligned}$$

which requires release height *h*, terminal velocity $v_T$, and wind speed $v_w$ (Nathan et al. 2011). For *A. syriaca*, we take $h = 0.866$ m and $v_T = 0.219$ m/s from Sullivan et al. (2018). We estimate wind speed using proxy data recorded by Seeley (2021) at Cedar Creek Ecosystem Science Reserve, which is 100 km from McKnight Prairie. We estimate $v_w = 4.23$ m/s, which is the maximum hourly speed over September 17–23 (a seed-release period used by Sullivan et al. (2018)) averaged over all 19 years with complete wind data. Equation (50) then yields $\mu = 16.7$ m, in reasonable agreement with the empirical mean 13.8 m measured by Platt and Weis (1977) at Cayler Prairie Preserve in Iowa.

With these parameters ($A = 137,000$ $\hbox {m}^2$, $L = 1,930$ m, $\mu = 16.7$ m), Theorem 7 gives the following upper bounds on seed loss probability:

$$\begin{aligned} p \le \frac{\mu L}{\pi A} = 7.5\% &  p \le \frac{\mu L}{2A} = 11.8\% \\ \text {(Symmetric)}\quad &  \text {(Asymmetric).}\quad \end{aligned}$$

These relatively low bounds are a result of the habitat’s large size, on the order of hundreds of meters, which gives a characteristic length scale *A*/*L* that is much greater than $\mu$. Even in the asymmetric case, $p \le 11.8\%$, meaning McKnight Prairie will retain almost 90% of *A. syriaca’s* seeds. While additional factors such as mortality rates within the patch may interact with seed loss to determine the ultimate outcome, the species’ high seed retention is encouraging.

**Fig. 6:**
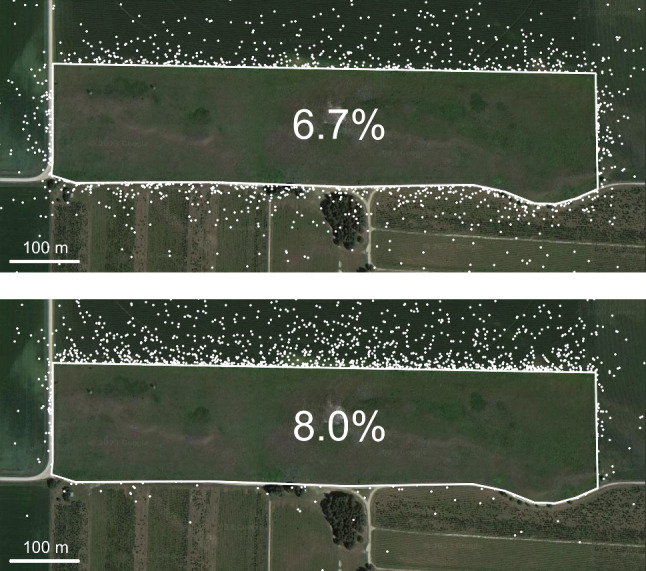
A random sample of several thousand *A. syriaca* seeds lost from McKnight Prairie (outlined in white) to the surrounding agricultural fields. More seeds are lost when dispersal is biased northward (bottom panel) than when dispersal is symmetric (top panel) even though mean dispersal distance is the same in both cases. Aerial image: Google, ©2023 Airbus, CNES/Airbus, Maxar Technologies, USDA/FPAC/GEO

### Simulation of Seed Loss

In addition to using Theorem 7 to derive upper bounds on seed loss from *A. syriaca* at McKnight prairie, we computed *p* using a numerical simulation consistent with the probabilistic definition of *p* in Eq. 9. We implemented a simple Monte Carlo style simulation in which a plant is placed uniformly at random within the habitat and a seed disperses from there, landing either inside or outside the habitat. This process was repeated 10 million times, and the fraction of lost seeds was reported as *p*. We ran the simulation twice, once for symmetric dispersal and once for asymmetric dispersal. In both cases, dispersal distance was sampled from an inverse Gaussian (Wald) distribution with mean $\mu =16.7$ m (as computed above) and shape parameter $\lambda = 2.5$ m (as reported in Sullivan et al. (2018)). In the symmetric case, the direction of the seed dispersal vector was chosen uniformly at random, but in the asymmetric case, a northern bias was introduced. To simulate a hypothetical prevailing northern wind, the direction, $\theta$, measured relative to due north, was sampled according to the probability density function $(1 + 10\theta ^2)^{-1}$ (normalized so that its integral from $-\pi$ to $\pi$ is 1). The results of the simulations were $p=6.7\%$ in the symmetric case and $p=8.0\%$ in the asymmetric case (Fig. 6). As expected, these values are less than the theoretical bounds of 7.5% and 11.8%.

**Fig. 7:**
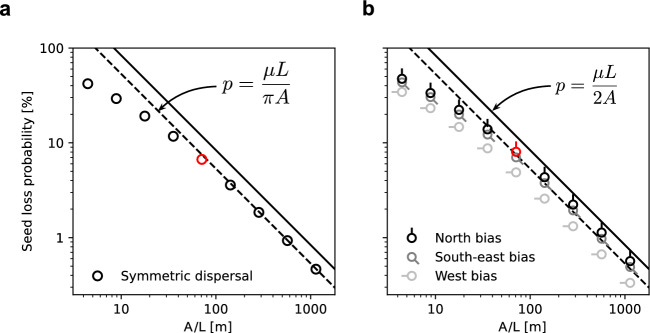
Impact of area to perimeter ratio *A*/*L*, on simulated seed loss probability *p*. Red symbols represent the two examples shown above, while other symbols represent hypothetical scenarios where McKnight Prairie is uniformly scaled up or down by a factor of 2, 4, 8, or 16, thus adjusting *A*/*L*. As the habitat grows larger, the symmetric bound (dashed line) becomes sharp, whereas the asymmetric bound (solid line) remains an over-estimate, especially for the alternative bias directions shown in gray (Color Figure Online)

### Comparison of Seed Loss Bounds to Simulations

To illustrate a wider range of seed loss scenarios, we also simulated the effect of increasing or decreasing the habitat’s area to perimeter ratio *A*/*L*, which is inversely proportional to the bounds on *p*. To change of *A*/*L*, we scaled the habitat size up or down uniformly. (Equivalently, we could have held the habitat size constant and scaled the mean dispersal distance $\mu$ down and up.) As shown in Fig. 7a, the simulated results trend closely with the theoretical bounds over a wide range of *A*/*L* values. For larger *A*/*L* values, the simulated seed loss probability for symmetric dispersal in particular shows remarkable agreement with the predicted bound. In these cases where the habitat is significantly larger than the length-scale of dispersal, $\mu L/\pi A$ becomes not only a bound but also a close estimate for *p*.

We suspect that this general assertion about the tightness of the symmetric bound could be proved, if formalized correctly—perhaps by generalizing the argument presented in Appendix B.2 that *p* approaches $\mu L/\pi A$ in a specific example. Such a formal proof would corroborate the related assertion by Chisholm and Lichstein (2009) that the immigration parameter, *m*, can be approximated by $\mu L/\pi A$ for large habitats.

In the case of asymmetric dispersal (Fig. 7b), the bound $\mu L/2A$ remains an overestimate of simulated seed loss even as *A*/*L* increases. The degree of overestimate depends on the bias direction of dispersal—for example, simulated seed loss is lower when dispersal is biased west, because fewer seeds are lost across the habitat’s long north and south edges.

## Discussion

Whereas numerical simulation of *p* requires detailed knowledge and coding of habitat geometry and plant dispersal kernels, the bounds of Theorem 7 require only mean dispersal distance $\mu$, habitat perimeter *L*, and habitat area *A*. We therefore anticipate that the bounds will provide an accessible upper estimate and worst-case-scenario indicator of seed loss across a variety of applications. For example, the value $1-p$ appears as the seed retention factor *f* in the spatially implicit model of colonization/extinction dynamics of Cooney et al. (2022) and as average dispersal success ${\bar{S}}$ in integrodifference models of spatial population dynamics (Van Kirk and Lewis 1997; Lutscher 2019). Further, *p* is equivalent to the immigration parameter *m* in the neutral theory of biodiversity (see Appendix A), for which the symmetric approximation is known (Chisholm and Lichstein 2009) but the asymmetric bound is new.

Because mean dispersal distance is the most challenging component of the bounds to estimate in practice, we discuss methods for doing so in Sect. 6.1 before outlining directions for future research in Sect. 6.2.

### Estimating Mean Dispersal Distance

Estimating mean dispersal distance is perhaps most straightforward for wind-dispersed seeds, for which mechanistic models are well-developed. In particular, the “ballistic” model

51 $$\begin{aligned} \mu =\frac{v_wh}{v_T} \end{aligned}$$

has long been used to estimate dispersal distance $\mu$ in terms of seed release height *h*, terminal velocity $v_T$, and wind speed $v_w$ (for a history, see Nathan et al. (2011)), and it remains the mean dispersal distance in the more sophisticated probabilistic WALD model that incorporates turbulent airflow (Katul et al. 2005). Both wind speed $v_w$ and seed release height *h* can be obtained from field measurements, while terminal velocity $v_T$ can be determined in a lab. Sullivan and colleagues (2018) describe measurement methods for these quantities and report values for 50 grassland species at Cedar Creek Ecosystem Science Reserve in Isanti County, Minnesota.

In the example in Sect. 5, estimating wind velocity was the most difficult part of using Eq. (51)—particularly matching wind data to the time and location of seed release. At the time of writing, the season of seed release was 5 months in the future and the height of wind sensors at the nearest weather stations was orders of magnitude higher than the height of seed release. To overcome such obstacles, one can use proxy measurements from similar sites (as we did) or wait for the season of seed release to take on-site field measurements.

Despite the challenges of estimating wind velocity as an input to the mechanistic model (51), this first-principles approach yielded an estimate of mean dispersal distance for *A. syriaca* that aligns well with the empricial observations of Platt and Weiss (1977). At the Cayler Prairie Preserve in Iowa, Platt and Weiss measured the distance travelled by 200 *A. syriaca* seeds that were released from naturally occurring heights in wind conditions of 10–15 km/hr. They computed a mean dispersal distance of 13.8 m ± 0.5 m standard error. Although this empirical estimate is lower than our model estimate of $\mu =16.7$ m, the difference is not great and might be due to different treatment of winds. The model’s proxy wind speed of 4.23 m/s translates to 15.2 km/h, which lies on the upper end of the conditions at Cayler Prairie Preserve. If instead we use the 10–15 km/hr wind range at Cayler Prairie Preserve as $v_w$, the model (51) predicts mean dispersal distances of 11.0 m $\le \mu \le 16.5$ m, and the empirical mean (13.8 m) falls squarely within this interval.

For seeds dispersed by mechanisms other than wind, existing literature offers several starting points for estimating mean dispersal distance $\mu$. Rough estimates can be obtained from a meta-analysis of 148 mostly empirical sources conducted by Thomson and colleagues (2011). In the course of studying relationships with plant height and seed mass, the authors condensed data on the mean dispersal distances of over 200 species into summary statistics grouped by 8 dispersal mechanisms (e.g. unassisted, water, vertebrate), presented in Appendix S3. Because the report does not provide data by species or list the primary research articles it draws upon, additional effort is required to narrow these order-of-magnitude bounds.

First, one can consult existing literature for proxy estimates. For example, Bullock and colleagues published a synthesis report in 2017 that lists dispersal data sources by species for 144 vascular plant species in Table S4 of the online supplement. Second, novel analyses can be performed to estimate dispersal distances. Methods for estimating dispersal across a variety of mechanisms are reviewed by Rogers et al. (2019). By considering a combination of dispersal estimates, one can develop a range that seems plausible for the species and site under consideration.

### Future Directions

As noted in Sect. 5.3, a salient area that remains to be explored is the conditions under which the quantities $\mu L/2A$ or $\mu L/\pi A$ serve not only as upper bounds but also as close approximations for *p*. Based on simulation data in Fig. 7, we expect the bound $\mu L/\pi A$ to be tight when dispersal is symmetric and mean dispersal distance is short relative to habitat size. Working numerically with square and circular patches, Chisholm and Lichstein (2009) find similar trends that are robust to different choices of dispersal kernel. Future work could pursue analytical convergence results and relax assumptions on patch shape.

One variable to consider while exploring tightness of bounds is the connectedness of habitat patches. Although our illustrations and example have featured a single connected habitat patch, the bounds also apply to a habitat “patch” consisting of multiple nearby components that are disconnected by roadways or other land uses. (In this case, the total perimeter and area of all components are summed to determine *L* and *A*, respectively.) Because dividing a habitat patch into two components via a narrow roadway may increase total perimeter *L* without substantially altering area or seed loss, the bounds of Theorem 7 may significantly overestimate seed loss for disconnected habitats. (Of course, a roadway could also alter dispersal patterns by changing animal behavior, wind, or water flow in ways that violate the assumptions of our model, as discussed in the next paragraph.)

Further theoretical efforts could work towards seed loss bounds that avoid one or several of the simplifying assumptions that underpin the dispersal model (9). First, we assume that the dispersal location random variable ${\textbf{X}}_d$ and plant location random variable ${\textbf{X}}_p$ are independent. However, dispersal patterns might vary according to plant location based on the effects of topographic slope and habitat edges on wind, water, or animal behavior. For example, in experimental patches of longleaf pine savanna embedded in a pine plantation matrix, Damschen and colleagues (2014) found significant differences in wind velocities and propagule dispersal patterns at various locations within a patch. Patch shape influenced this phenomenon, with long corridors of savanna in particular promoting uplifting and long distance dispersal. Second, we assume that plant locations ${\textbf{X}}_p$ follow a uniform distribution within a habitat, but variations in local site conditions could bias the plant distributions. In keeping with the orientation of this work towards estimates that use minimal information inputs, it would be particularly interesting to find bounds that allow for the variations described above without requiring their full characterization.

Lastly, we note that the binary classification of land as habitable or not habitable may be unrealistic for some ecosystems. Indeed, dispersal out of targeted reserves can have positive spillover effects on the surrounding landscape (Brudvig et al. 2009), pointing towards a more nuanced interpretation of seed loss from a habitat patch. Combining estimates of seed loss with estimates of establishment in the surrounding matrix (e.g. Craig et al. (2011)) could yield insights into the potential benefits of dispersal out of a reserve.

## Figures and Tables

**Fig. 8:**
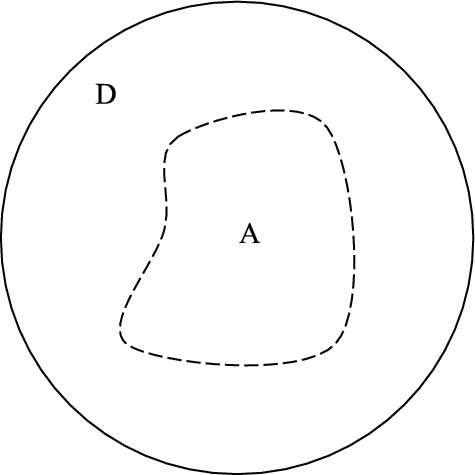
The quadrat A is embedded in a large but finite disk D, which approximates an infinite surrounding landscape

**Fig. 9:**
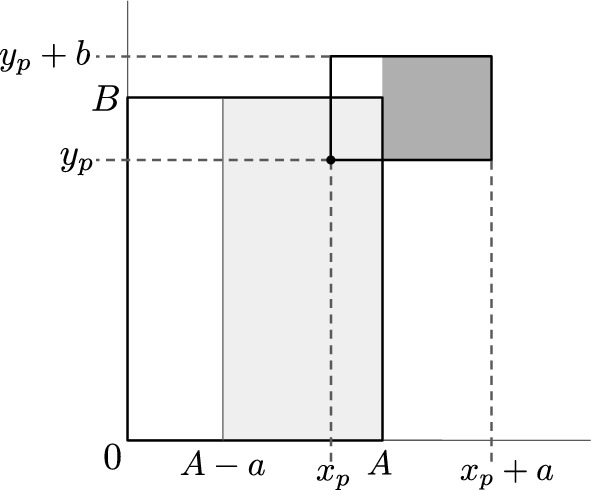
Quantities used to bound the integral (B28). The light and dark grey rectangles represent the domains of integration for the outer and inner double integrals, respectively (Color Figure Online)

**Fig. 10:**
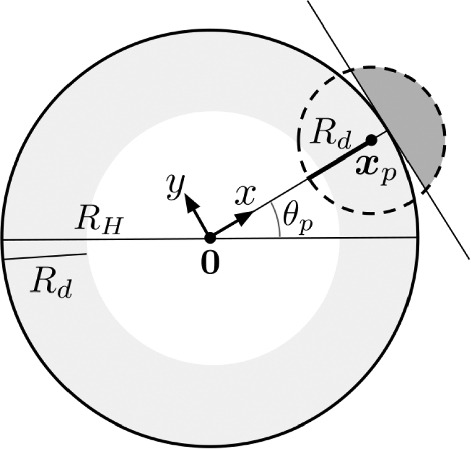
Quantities and coordinates used in the proof of Proposition 9

**Fig. 11:**
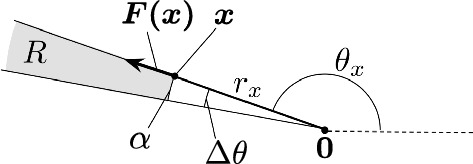
Notation used in the construction of the vector field ${\textbf{F}}({\varvec{x}})$

## Data Availability

Our estimate of *A. syriaca*’s mean dispersal distance (see Eq. (50)) is based on plant height and seed terminal velocity data measured by Sullivan et al. (2018), as well as hourly wind speed data from Seeley (2021). The satellite image of McKnight Prairie used in Fig. 6 is from Google Maps (2024).

## Appendix A: Equivalence of Seed Loss Probability and Immigration Parameter

Here we demonstrate that the immigration parameter *m* formalized by Chisholm and Lichstein (2009) in the context of the neutral theory of biodiversity is equivalent to the seed loss probability *p* defined by Eq. (9).

Chisholm and Lichstein imagine “a spatially homogeneous infinite two-dimensional landscape” (p. 1386) occupied by sessile organisms at a constant density. All individuals generate propagules at the same rate, and all propagules share the same rotationally symmetric dispersal kernel. Within the landscape, an arbitrary spatial community (or quadrat) A with area *A* is chosen. The immigration parameter *m* associated with A is defined as

A1 $$\begin{aligned} m=\frac{1}{A}\iint _{\text {A}} m_{x,y} \, dxdy \end{aligned}$$

where $m_{x,y}$ is “the probability that the replacement individual at location (*x*, *y*) is drawn from outside the local community” (p. 1386). If opportunities for replacement occur evenly within the community, then *m* gives the community-wide probability that this replacement comes externally.

In Chisholm and Lichstein’s landscape, all dispersal events follow the same probability distribution. Let the random variable *Q* denote the source of a propagule (seed), with probability density function $f_Q$. Given Chisholm and Lichstein’s assumptions of spatial homogeneity, *Q* should follow a uniform distribution over the landscape. However, a uniform distribution can be defined only on a finite domain. We therefore initially restrict propagule sources to a large but finite disk D $\supset$ A of area *D* within the landscape, as shown in Fig. 8, so $f_Q(q)=1/D$. (Later, we take the limit as D extends to all of ${\mathbb {R}}^2$.)

Let the random variable *S* denote the landing location of a seed, with probability density function $f_S$. We will show later that as D becomes large $f_S(s)$ approaches 1/*D* for any fixed *s*.

Denote the joint distribution of *Q* and *S* as $f_{Q,S}(q,s)$. Then the conditional probability density functions are defined as

A2 $$\begin{aligned} f_{S|Q=q}(s)&=\frac{f_{Q,S}(q,s)}{f_Q(q)} \end{aligned}$$

A3 $$\begin{aligned} \text {and} \quad f_{Q|S=s}(q)&=\frac{f_{Q,S}(q,s)}{f_S(s)}. \end{aligned}$$

The conditional density $f_{S|Q=q}(s)$ reflects the probability density of a seed sourced from a fixed location *q* landing at *s*, while the conditional density $f_{Q|S=s}(q)$ reflects the probability density that a seed landing at a fixed location *s* came from *q*.

With a slight change in notation, we rewrite $m_{x,y}$ as $m_s$ and change the quadrat name A to the patch name *H*. Then

A4 $$\begin{aligned} m_s=\iint \limits _{q\in \text {D}\setminus H} f_{ Q | S=s}(q) \, dA. \end{aligned}$$

We average $m_s$ over the patch (quadrat) *H* to get

A5 $$\begin{aligned} m&= \frac{1}{A}\iint \limits _{s\in H} \left( \ \iint \limits _{q\in \text {D}\setminus H} f_{ Q | S=s}(q) \, dA \right) \, dA \end{aligned}$$

A6 $$\begin{aligned}&=\frac{1}{A}\iint \limits _{s\in H} \left( \ 1 - \iint \limits _{q\in H} f_{ Q | S=s}(q) \, dA \right) \, dA \end{aligned}$$

A7 $$\begin{aligned}&= 1 - \frac{1}{A}\iint \limits _{s\in H} \left( \ \iint \limits _{q\in H} f_{ Q | S=s}(q) \, dA \right) \, dA. \end{aligned}$$

The continuous version of Bayes’ Theorem gives that

A8 $$\begin{aligned} f_{ Q | S=s}(q)=\frac{f_{S|Q=q}(s) f_Q(q)}{f_S(s)}, \end{aligned}$$

so

A9 $$\begin{aligned} m&= 1 - \frac{1}{A}\iint \limits _{s\in H} \left( \ \iint \limits _{q\in H} f_{S|Q=q}(s) \cdot \frac{f_Q(q)}{f_S(s)} \, dA \right) \, dA. \end{aligned}$$

Since the conditional density $f_{S|Q=q}(s)$ in Eq. (A9) describes the probability that a seed lands at location *s* given that it originated from a parent at location *q*, it is equivalent to the dispersal kernel $f_d$ of this work evaluated at the vector $s-q$. Hence

A10 $$\begin{aligned} m&= 1 - \frac{1}{A}\iint \limits _{s\in H} \left( \ \iint \limits _{q\in H} f_d(s-q) \cdot \frac{f_Q(q)}{f_S(s)} \, dA \right) \, dA. \end{aligned}$$

We now argue that the fraction $\frac{f_Q(q)}{f_S(s)}$ in Eq. (A10) approaches 1 as the disk D approaches ${\mathbb {R}}^2$. We have that

A11 $$\begin{aligned} S=Q+X_d \end{aligned}$$

where the vector $X_d$ is the dispersal random variable with probability density function $f_d$. Because dispersal probabilities are independent of seed source, it follows (see Eq. (11)) that

A12 $$\begin{aligned} f_S(s)&=\iint \limits _{q\in {\mathbb {R}}^2}f_Q(q)f_d(s-q) \, dA \end{aligned}$$

A13 $$\begin{aligned}&=\iint \limits _{q\in \text {D}}\frac{1}{D}f_d(s-q) \, dA \end{aligned}$$

A14 $$\begin{aligned}&=\frac{1}{D}\iint \limits _{q\in \text {D}}f_d(s-q) \, dA. \end{aligned}$$

In the limit as the radius of the disk D approaches infinity, we have

A15 $$\begin{aligned} \iint \limits _{q\in \text {D}}f_d(s-q) \, dA \rightarrow 1, \end{aligned}$$

so

A16 $$\begin{aligned} \frac{f_Q(q)}{f_S(s)}=\frac{1/D}{(1/D) \iint \limits _{q\in \text {D}}f_d(s-q) \, dA} \rightarrow 1. \end{aligned}$$

Thus, after taking the limit as a disk of uniformly distributed plants approaches ${\mathbb {R}}^2$,

A17 $$\begin{aligned} m&= 1 - \frac{1}{A}\iint \limits _{s\in H} \left( \ \iint \limits _{q\in H} f_d(s-q) \, dA \right) \, dA. \end{aligned}$$

Interchanging the order of integration over *H*, we have

A18 $$\begin{aligned} m&= 1 - \frac{1}{A}\iint \limits _{q\in H} \left( \ \iint \limits _{s\in H} f_d(s-q) \, dA \right) \, dA \end{aligned}$$

A19 $$\begin{aligned}&= \frac{1}{A}\iint \limits _{q\in H} \left( \ 1 - \iint \limits _{s\in H} f_d(s-q) \, dA \right) \, dA \end{aligned}$$

A20 $$\begin{aligned}&= \frac{1}{A}\iint \limits _{q\in H} \left( \ \iint \limits _{s\in H^c} f_d(s-q) \, dA \right) \, dA \end{aligned}$$

and renaming the variable *q* as ${\varvec{x}}_p$ and the variable *s* as ${\varvec{x}}$ yields

A21 $$\begin{aligned} m&= \frac{1}{A}\iint \limits _{{\varvec{x}}_p\in H} \left( \ \iint \limits _{{\varvec{x}}\in H^c} f_d({\varvec{x}}-{\varvec{x}}_p) \, dA \right) \, dA \end{aligned}$$

A22 $$\begin{aligned}&= p \end{aligned}$$

as desired (see Proposition 1).

## Appendix B: Sharpness of Bounds

### B.1: Asymmetric Dispersal

In Theorem 7 we prove that, without any assumptions on the symmetry of the dispersal kernel, the proportion *p* of seeds lost from a habitat patch with area *A* and perimeter *L* is bounded by

B23 $$\begin{aligned} p\le \frac{\mu L}{2A}, \end{aligned}$$

where $\mu$ is the mean dispersal distance of the seeds. Rearranging, this is equivalent to

$$\begin{aligned} \frac{pA}{\mu L}\le \frac{1}{2}. \end{aligned}$$

Let $k=\dfrac{pA}{\mu L}$, so that the bound (B23) is equivalent to $k\le 1/2$. The following proposition implies that this bound is the lowest possible under the assumptions given in Sect. 2.

#### Proposition 8

There exist habitat geometries and dispersal kernels that make $k\equiv \dfrac{pA}{\mu L}$ arbitrarily close to 1/2.

#### Proof

Let $a<A$ and $b<B$ be positive real numbers. Consider a rectangular habitat

$$\begin{aligned} H=\{(x,y)\in {\mathbb {R}}^2 \ | \ 0<x<A \text { and } 0<y<B\}, \end{aligned}$$

and a dispersal kernel

$$\begin{aligned} f_d(x,y)={\left\{ \begin{array}{ll} \frac{1}{ab} & 0< x< a \text { and } 0< y < b \\ 0 & \text {otherwise}. \end{array}\right. } \end{aligned}$$

We bound the factors in *k* in terms of *a*, *b*, *A*, and/or *B*. First, we have the equality

B24 $$\begin{aligned} L=2(A+B). \end{aligned}$$

Second, from the definition (7) of mean dispersal distance we have

B25 $$\begin{aligned} \begin{aligned} \mu&=\iint \limits _{{\mathbb {R}}^2}|{\varvec{x}}_d|f_d({\varvec{x}}_d)\, dA\\&=\int \limits _0^b\int \limits _0^a \sqrt{x^2+y^2}\frac{1}{ab}\,dx\,dy\\&\le \int \limits _0^b\int \limits _0^a (x+y)\frac{1}{ab}\,dx\,dy\\&=\frac{a+b}{2}. \end{aligned} \end{aligned}$$

From (B24) and (B25) it follows that

B26 $$\begin{aligned} k\ge \frac{pA}{\left( \frac{a+b}{2}\right) \cdot 2(A+B)}=\frac{pA}{(a+b)(A+B)}. \end{aligned}$$

Next we bound *pA*, which from Proposition 1 is equivalent to the integral

B27 $$\begin{aligned} pA=\iint \limits _{{\varvec{x}}_p\in H} \ \ \iint \limits _{{\varvec{x}}\in H^c} f_d({\varvec{x}}- {\varvec{x}}_p) \, dA \, dA. \end{aligned}$$

By restricting the domains of integration in Eq. (B27), we can only decrease the integral of the non-negative quantity $f_d$. Let ${\varvec{x}}_p=(x_p,y_p)$. Choosing bounds for ${\varvec{x}}$ and ${\varvec{x}}_p$ over which $f_d({\varvec{x}}-{\varvec{x}}_p)= 1/ab$ (see Fig. 9), we obtain

B28 $$\begin{aligned} pA&\ge \int \limits _0^B\int \limits _{A-a}^{A}\left( \int \limits _{y_p}^{y_p+b}\int \limits _A^{x_p+a}\frac{1}{ab}\,dx\,dy\right) dx_p\,dy_p \end{aligned}$$

B29 $$\begin{aligned}&=\int \limits _0^B\int \limits _{A-a}^{A}\left( \frac{x_p+a-A}{a} \right) dx_p\,dy_p \end{aligned}$$

B30 $$\begin{aligned}&=\frac{B}{a} \int \limits _{A-a}^{A}(x_p+a-A) \,dx_p, \end{aligned}$$

which simplifies to

B31 $$\begin{aligned} pA\ge \frac{aB}{2}. \end{aligned}$$

Combining inequalities (B26) and (B31) yields

$$\begin{aligned} k\ge \frac{aB}{2(a+b)(A+B)}. \end{aligned}$$

For habitats with $B>>A$ the ratio $\dfrac{B}{A+B}$ can be made arbitrarily close to 1. Similarly, for dispersal kernels with $a>>b$, the ratio $\dfrac{a}{a+b}$ can also be made arbitrarily close to 1. It follows that *k* can be made arbitrarily close to $\frac{1}{2}$. $\square$

Thus, no values below 1/2 suffice to bound *k* from above. It is interesting to note that the ultimate quantities compared in this argument concern the aspect ratio of the rectangular habitat *H* and the rectangular support for the dispersal kernel $f_d$. In particular, we imagine the aspect ratios becoming extreme in orthogonal directions.

### B.2: Symmetric Dispersal

Following the approach in Appendix B.1, we define

B32 $$\begin{aligned} k=\frac{pA}{\mu L} \end{aligned}$$

so that the existence of habitat geometries and dispersal kernels that make *k* arbitrarily close to $1/\pi$ implies that *p* can be arbitrarily close to its bound $\dfrac{\mu L}{\pi A}$ in Theorem 7 when dispersal is rotationally symmetric.

#### Proposition 9

There exist habitat geometries and rotationally symmetric dispersal kernels that make $k\equiv \dfrac{pA}{\mu L}$ arbitrarily close to $1/\pi$.

#### Proof

Define a disk habitat *H* of radius $R_H$; that is,

$$\begin{aligned} H=\{{\varvec{x}}: \ |{\varvec{x}}|<R_H\}, \end{aligned}$$

and take the dispersal kernel $f_d$ to be a uniform distribution over a disk support with radius $R_d$:

$$\begin{aligned} f_d({\varvec{x}})={\left\{ \begin{array}{ll} \dfrac{1}{\pi R_d^2} & \text {if }|{\varvec{x}}|<R_d\\ 0 & \text {otherwise}. \end{array}\right. } \end{aligned}$$

Simple geometry gives that

B33 $$\begin{aligned} L=2\pi R_H. \end{aligned}$$

And by definition we have

B34 $$\begin{aligned} \begin{aligned} \mu&=\iint \limits _{{\mathbb {R}}^2} |{\varvec{x}}|f_d({\varvec{x}}) \, dA\\&=\int \limits _0^{2\pi }\int \limits _0^{R_d} r \frac{1}{\pi R_d^2} r \, dr \, d\theta \\&=\frac{2}{3}R_d. \end{aligned} \end{aligned}$$

Substituting for *pA*, *L*, and $\mu$ in Eq. (B32) using Proposition 1 and Eqs. (B33) and (B34) yields

$$\begin{aligned} k=\frac{3}{4\pi R_d R_H} \iint \limits _{{\varvec{x}}_p\in H} \ \ \iint \limits _{{\varvec{x}}\in H^c} f_d({\varvec{x}}- {\varvec{x}}_p) \, dA \, dA. \end{aligned}$$

Because the only plant positions ${\varvec{x}}_p$ that contribute to seed loss are those at distance between $R_H-R_d$ and $R_H$ from the origin, we express the outer double integral in polar coordinates as

B35 $$\begin{aligned} k=\frac{3}{4\pi R_d R_H} \int \limits _0^{2\pi } \int \limits _{R_H-R_d}^{R_H} \left( \ \ \iint \limits _{{\varvec{x}}\in H^c} f_d({\varvec{x}}- {\varvec{x}}_p) \, dA \right) \, r_p \, dr_p \, d\theta \end{aligned}$$

where ${\varvec{x}}_p=(r_p,\theta _p)$. We use Cartesian coordinates ${\varvec{x}}=(x,y)$ for the inner double integral, with the *x*-axis aligned with the vector ${\varvec{x}}_p$ (see Fig. 10).

To simplify computations we restrict the domain of the inner integral to the segment of the circle of radius $R_d$ centered on ${\varvec{x}}_p$ that is cut by the chord tangent to the habitat boundary at the *x*-axis. Then

B36 $$\begin{aligned} k\ge \frac{3}{4\pi R_d R_H} \int \limits _0^{2\pi } \int \limits _{R_H-R_d}^{R_H} \int \limits _{R_H}^{r_p+R_d} \int \limits _{-\sqrt{R_d^2-(x-r_p)^2}}^{\sqrt{R_d^2-(x-r_p)^2}} \ \frac{1}{\pi R_d^2} \ dy \, dx \, r_p \, dr_p \, d\theta . \end{aligned}$$

The $\theta$ and *y* integrals can be evaluated directly, yielding

B37 $$\begin{aligned} k&\ge \frac{3}{4\pi R_d R_H} 2\pi \int \limits _{R_H-R_d}^{R_H} \int \limits _{R_H}^{r_p+R_d} 2\sqrt{R_d^2-(x-r_p)^2}\frac{1}{\pi R_d^2}\, dx \, r_p \, dr_p \end{aligned}$$

B38 $$\begin{aligned}&=\frac{3}{\pi R_d^3 R_H}\int \limits _{R_H-R_d}^{R_H} \int \limits _{R_H}^{r_p+R_d} \sqrt{R_d^2-(x-r_p)^2} \ dx \, r_p \, dr_p. \end{aligned}$$

Because $r_p>R_H-R_d$ over the domain of integration, one can eliminate the factor of $r_p$ from the integrand as follows:

B39 $$\begin{aligned} k\ge \frac{3(R_H-R_d)}{\pi R_d^3 R_H}\int \limits _{R_H-R_d}^{R_H} \int \limits _{R_H}^{r_p+R_d} \sqrt{R_d^2-(x-r_p)^2} \ dx \, dr_p. \end{aligned}$$

We first make the substitution $u=x-r_p$ and then reverse the order of integration:

B40 $$\begin{aligned} k&\ge \frac{3(R_H-R_d)}{\pi R_d^3 R_H}\int \limits _{R_H-R_d}^{R_H} \int \limits _{R_H-r_p}^{R_d} \sqrt{R_d^2-u^2} \ du \, dr_p \end{aligned}$$

B41 $$\begin{aligned}&=\frac{3(R_H-R_d)}{\pi R_d^3 R_H}\int \limits _{0}^{R_d} \int \limits _{R_H-u}^{R_H} \sqrt{R_d^2-u^2} \ dr_p \, du \end{aligned}$$

B42 $$\begin{aligned}&=\frac{3(R_H-R_d)}{\pi R_d^3 R_H}\int _{0}^{R_d} u \sqrt{R_d^2-u^2} \ du. \end{aligned}$$

Evaluating the remaining integral yields $R_d^3/3$, and so

B43 $$\begin{aligned} k\ge \frac{R_H-R_d}{\pi R_H}. \end{aligned}$$

By taking $R_H>>R_d$, *k* can be made arbitrarily close to $1/\pi$, as desired. $\square$

## Appendix C: Discussion of F

For the purpose of proving Theorem 7, the vector field ${\textbf{F}}$ was a convenient mathematical stepping stone tied to the idea of seed flow to build intuition. But a more precise physical interpretation is worth noting. Specifically, *the flux of* ${\textbf{F}}$ *across a curve can be interpreted as the probability that a given seed would pass through that curve if all seeds traveled in straight lines*. Here, we show that this physical description of ${\textbf{F}}$ leads logically to its mathematical definition in Eq. (13).

Consider a single plant at the origin with dispersal kernel $f_d$. Given a location ${\varvec{x}}$ in the landscape with polar coordinates $(r_x,\theta _x)$, we define an arc $\alpha$ starting at ${\varvec{x}}$ and subtending an angle $\Delta \theta$. According to our interpretation above, the flux of ${\textbf{F}}$ across $\alpha$ must equal the probability that a seed passes through $\alpha$. Since we are working under the hypothetical assumption that seeds travel in straight lines, the seeds that pass through $\alpha$ are exactly those that land in the infinite polar rectangle $R=\{(r,\theta ) \ | \ r\ge r_x, \, \theta _x\le \theta \le \theta _x+\Delta \theta \}$ shown in Fig. 11. Thus,

C44 $$\begin{aligned} \hbox {flux of} \,{\textbf{F}}\, \hbox {across}\, \alpha = P({\textbf{X}}_d\in R) = \int \limits _{\theta _x}^{\theta _x+\Delta \theta }\int \limits _{r_x}^\infty f_d(r,\theta ) \, r \, dr \, d\theta . \end{aligned}$$

To be consistent with the straight-line dispersal hypothetical, we choose ${\textbf{F}}$ to point radially outward from the origin. Then since ${\textbf{F}}$ is normal to $\alpha$, its flux across $\alpha$ can also be expressed as

C45 $$\begin{aligned} \hbox {flux of}\, {\textbf{F}}\,\hbox {across} \,\alpha = \int \limits _{\theta _x}^{\theta _x+\Delta \theta } |{\textbf{F}}(r_x, \theta )| r_x \, d\theta , \end{aligned}$$

where $r_x \, d\theta$ serves as the differential arc length *ds*. After combining Eqs. (C44) and (C45), we may shed the $\theta$ integrals by dividing by $\Delta \theta$ and then taking the limit as $\Delta \theta$ approaches zero, leaving

C46 $$\begin{aligned} |{\textbf{F}}({\varvec{x}})|r_x = \int \limits _{r_x}^\infty f_d(r,\theta _x) \, r \, dr. \end{aligned}$$

Solving for $|{\textbf{F}}({\varvec{x}})|$ and then multiplying by the unit vector ${\varvec{x}}/r_x$ to recover ${\textbf{F}}$’s direction gives our definition:

$$\begin{aligned} {\textbf{F}}({\varvec{x}}) = \frac{{\varvec{x}}}{r_x^2}\int \limits _{r_x}^\infty f_d(r,\theta _x) \, r \, dr. \end{aligned}$$

## Code Availability

Code that (i) averages wind speed from Cedar Creek data, (ii) measures McKnight area and perimeter from Google photo, and (iii) simulates seed loss is available at github.com/BenHafner/SeedLoss.
